# Construction and Chemical Profile on “Activity Fingerprint” of Citri Reticulatae Pericarpium from Different Cultivars Based on HPLC-UV, LC/MS-IT-TOF, and Principal Component Analysis

**DOI:** 10.1155/2020/4736152

**Published:** 2020-03-03

**Authors:** Guodong Zheng, Yingxin Chao, Meixia Luo, Bin Xie, Dedong Zhang, Pingjun Hu, Xiujuan Yang, Depo Yang, Minyan Wei

**Affiliations:** ^1^Key Laboratory of Molecular Target & Clinical Pharmacology and the State Key Laboratory of Respiratory Disease, School of Pharmaceutical Sciences & The Fifth Affiliated Hospital, Guangzhou Medical University, Guangzhou, China; ^2^The Affiliated Brain Hospital of Guangzhou Medical University, Guangzhou Huiai Hospital, Guangzhou, China; ^3^Tianda Pharmaceutical (Zhuhai) Co., Ltd., Zhuhai, China; ^4^School of Pharmaceutical Sciences, Sun Yat-sen University, Guangzhou, China

## Abstract

Citri Reticulatae Pericarpium (CRP), known as Chenpi (CP) in Chinese, is a medicinal food for health and fitness. In order to find out the characteristic activity chemicals distinguishing various cultivars of CRP and provide a reference for effective development of citrus resources, an “activity fingerprint” of CRP from 21 different cultivars was established based on the evaluation of antitussive and expectorant activities. There were 18 common peaks in the HPLC fingerprint, of which 3 flavonoid glycosides and 14 polymethoxyflavonoids (PMFs) were identified by LC/MS-IT-TOF. Furthermore, five characteristic chemicals were determined and similarity calculation with principal component analysis (PCA) was combined together to compare the similarity and difference among various cultivars. The results showed that some other cultivars were also similar to *C*. *reticulata* “Chachi” except for *C*. *reticulata* “Tangerina” and *C*. *reticulata* “Dahongpao” recorded in Chinese Pharmacopoeia. Most importantly, the peels of *C*. *reticulata* “Shiyueju,” *C*. *reticulata* “Ponkan,” *C*. *reticulata* “Tribute,” and *C*. *reticulata* “Bayueju,” traditionally rarely used for medicinal food, were highly similar to that of *C*. *reticulata* “Chachi” and rich in bioactive flavonoids, which can be considered the effective medicinal resources of CRP.

## 1. Introduction

Citri Reticulatae Pericarpium (CRP), also known as Chenpi (CP) in Chinese, the dry mature peel of *Citrus reticulata* Blanco or its cultivated varieties [1], has been traditionally used as both nutritional food and herbal medicine for clinic treatment of cough with phlegm, vomiting and diarrhea, and stomach ache [2]. There are four cultivars of CRP officially listed in Chinese Pharmacopoeia (2015 version), such as *C*. *reticulata* “Chachi,” *C*. *reticulata* “Dahongpao,” *C*. *reticulata* “Tangerina,” and *C*. *reticulata* “Unshiu.” Among them, the desiccative ripe peel of *Citrus reticulata* “Chachi,” called Guang Chenpi (GCP) in Chinese and produced chiefly in Xinhui (Guangdong, China), is well known as a traditional genuine herb with high quality. However, there are no more uniform quality standards on CRP application and other nonlisted cultivars were often sold at best quality prices, resulting in a confusing market with uneven quality of CRP. Thus, it is necessary to establish a scientific method and find the characteristic activity chemicals distinguishing various cultivars of CRP for quality control.

High-performance liquid chromatography (HPLC) fingerprint analysis has recently been applied as a quality evaluation method with high specificity, stability, reproducibility, and integrity because a large number of samples could be well analyzed by describing the similarities and differences through similarity results [3, 4]. Although it is a good assumption to evaluate and compare a great number of CRP samples through the HPLC fingerprint, previous studies mainly focused on a single cultivar of *C*. *reticulata* “Chachi” from different places of Guangdong Province or four recorded cultivars [5], while other nonrecorded but common cultivars have not been taken into consideration. It is more reliable to find out characteristics distinguishing varieties to protect the health of the CRP market when adopting as many different cultivars as possible for quality comparison. In addition, the HPLC fingerprint is just a kind of qualitative analysis and the compounds corresponding to its characteristic peak have not been profiled [6], so it is of significance to further study the characteristic components in combination with other identification and quantification techniques.

Flavonoids have been proved to be the main chemical components in CRP, extracted mostly from methanol, and distributed into two types: flavonoid glycosides and polymethoxyflavonoids (PMFs). And recently, pharmacological studies reported for individual flavonoids described anti-inflammatory [7, 8], antitumor [9, 10], antibacterial [11, 12], and antioxidant activities [13, 14] and cardiovascular protection [15, 16]. However, there are few reports about the pharmacological activity of the mixed CRP extract so that the HPLC fingerprint has not yet been proved to relate to active compounds. Moreover, it is uncertain whether the methanol extract of CRP (MECRP) has antitussive and expectorant activities. The antitussive and expectorant activity evaluation of the MECRP played an indispensable part of “activity fingerprint” for systematical quality control.

In this study, 88 samples including 21 different cultivars were used for quality analysis. The MECRP was firstly prepared on the animal models for antitussive and expectorant activity evaluation. HPLC and HPLC combined with ion-trap and time-of-flight mass spectrometry (LC/MS-IT-TOF) were then applied to clarify the characteristic chemicals corresponding to each common peak in the fingerprint and determine the content of five main flavonoids. Finally, similarity calculation and principal component analysis (PCA) were combined to compare the similarity and difference among various cultivars. Overall, this effective method of “activity fingerprint” would not only contribute to establishing an excellent quality evaluation system of CRP to find out the characteristic activity chemicals distinguishing various cultivars but also provide a reference for the effective development of citrus resources.

## 2. Materials and Methods

### 2.1. Plant and Animal Materials

Eighty-eight batches of samples including 21 different cultivars were gathered from different major citrus-producing regions in China (Table 1). The sample S1 (Lot no. 20141016) is chosen as reference crude herb for the activity assay and method validation with the qualification of China's protected geographical indication product, provided by the natural planting base in Luokeng Town by Xinbaotang Company. The voucher specimens, appraised by Prof. Bo Wu, have been deposited at the Laboratory of Pharmacognosy, Guangzhou Medical University, Guangdong Province, China.

SPF Kunming mice weighing 18 to 22 g, half male and female, were provided by the Medical Experimental Animal Center of Guangdong Province (License SCXK[Yue] 2013-0002). Animals were fasted for 12 hours before the first and the final administration but given water ad libitum. All the experimental procedures were performed in accordance with the Regulation on the Administration of Laboratory Animals issued by the State Council of China. All animals were killed by cervical dislocation, and all efforts were made to minimize suffering.

### 2.2. Chemicals and Reagents

The positive control drug, dextromethorphan hydrobromide (DH) tablets, for the antitussive activity assay was purchased from Baiyunshan Guanghua (China). The ambroxol hydrochloride (AH) tablets, used for the expectorant activity assay, were obtained from Boehringer Ingelheim (Shanghai, China). The solvents for HPLC and LC/MS-IT-TOF analysis, HPLC-grade acetonitrile and purified water, used after filtration through membranes, were procured from Honeywell (America) and Yibao (China), respectively. Hesperidin (Chengdu Must Bio-Technology, Chengdu, China), nobiletin (Chengdu Must Bio-Technology, Chengdu, China), 3,5,6,7,8,3′,4′-heptamethoxyflavone (Weikeqi, Sichuan, China), tangeretin (Chengdu Must Bio-Technology, Chengdu, China), and 5-hydroxy-6,7,8,3′,4′-pentamethoxyflavone (Spring & Autumn, Nanjing, China) were all of reference standard grade, with the high purity above 98%. All other reagents were of analytical grade and pure.

### 2.3. Standard Solution and MECRP Preparation

Each reference of the five flavonoids was dissolved together in methanol at an appropriate concentration to obtain the mixed standard solution. Each sample powder was extracted ultrasonically with 100 times of methanol (w/v) for 30 minutes at 320 W [17]. The solvent of the sample S1 was eliminated by a rotary evaporator to obtain the dried MECRP (extraction rate = 31%, g/g), which was dispersed to different concentrations with distilled water for the activity assay. Meanwhile, all the sample solutions with the MECRP were filtered through a 0.22 *μ*m membrane for HPLC-UV and LC/MS-IT-TOF analysis.

### 2.4. Evaluation of Antitussive and Expectorant Activities of MECRP

#### 2.4.1. Antitussive Activity Assay

Fifty Kunming mice were randomly allocated to five groups of half male and half female each: control, MECRP-20, MECRP-40, MECRP-80, and DH-60. The control group and standard group received normal saline (0.9%) and DH (60 mg/kg) daily for 7 continuous days, respectively, while the test groups were administered orally with the MECRP (20, 40, and 80 mg/kg). After final administration for an hour, each animal was put in a sealed glass container and exposed to 25% ammonia water aerosol for 20 s by an ultrasonic nebulizer [18]. The mice were then removed to observe the incubation period of cough and bouts of cough within 3 minutes.

#### 2.4.2. Expectorant Activity Assay

Kunming mice were divided into five groups (*n* = 10/group): control, MECRP-20, MECRP-40, MECRP-80, and AH-40, and each of the groups was medicated orally continuously for 7 days (1 time/day). The control group and treated groups were administrated based on the method of the antitussive activity assay, whereas the standard group received AH 40 mg/kg orally. Thirty minutes after the final administration, each mouse was injected intraperitoneally with 0.5 mL phenol red solution in saline and killed by cervical dislocation 30 minutes later. The tracheas of the same length were taken from the thyroid cartilage to the trachea branch of the mice and washed ultrasonically with 3 mL NaOH solution (0.03 mol/L) in saline for 10 minutes [19]. Moreover, the absorbance of phenol red in the solution was measured spectrophotometrically at a wavelength of 546 nm, and its content was calculated under the linear regression equation *y* = 0.1428*x* + 0.0062 (*R*^2^ = 0.9993) (“x” denotes the concentration of phenol red solution and “y” denotes the absorbance).

#### 2.4.3. Statistical Analysis

The results of activity assays were statistically analyzed by SPSS (Version 16.0) and GraphPad Prism software (Version 5.0), which are expressed as mean ± standard deviation (SD). One-way ANOVA was used for the data analysis, followed by the LSD test to compare the differences between control and test groups. *P* < 0.05 was considered statistically significant in this study.

### 2.5. HPLC Fingerprint Analysis

The HPLC condition of the MECRP was performed in a previous method described by Luo et al. [17]. SIL-20A HPLC (Shimadzu, Japan) equipped with an SPD-20A UV detector and a Diamonsil C18 column (250 × 4.6 mm, i.d. 5 *μ*m) was used for HPLC analysis. The mobile phase was solution A (water) and solution B (acetonitrile) with the following gradient program: 0–15 min, 15–40% B; 15–35 min, 40–50% B; 35–40 min, 50–75% B; and 40–50 min, 75–85% B. Other HPLC conditions are shown as follows: flow rate: 1.0 mL/min; column temperature: 25°C; injection volume: 20 *μ*L; and detection wavelength: 283 nm and 330 nm. The chromatograms (330 nm) of *C*. *reticulata* “Chachi” samples produced in Xinhui (S1–S12) were firstly imported into the software “Similarity Evaluation System for Chromatographic Fingerprint of Traditional Chinese Medicine (Version 2012)” to establish the GCP fingerprint. With the median method and choosing three chromatographic peaks of hesperidin, nobiletin, and tangeretin for multipoint calibration, a GCP common pattern was generated. Next, the final fingerprint was established according to the representative chromatograms of remaining cultivars (S13–S88) together with the GCP common pattern. The similarity values of different cultivars were finally calculated based on the GCP common pattern.

### 2.6. LC/MS-IT-TOF Analysis

LC/MS-IT-TOF analysis was carried out as previously reported with a minor modification [20]. The HPLC instrument equipped with the ion-trap and time-of-flight mass spectrometer (Shimadzu, Japan) through electrospray ionization (ESI) was used. The compounds of extraction were isolated from the HPLC system with the same condition as in HPLC analysis mentioned above. The electrospray ionization (ESI) source was operated in a positive ionization mode, and its conditions are listed as follows: nitrogen gas flow, 1.5 L/min; dry gas temperature, 200°C; fragmentation voltage, 1.62 kV; nebulizer pressure, 45 psi; and full and dependent scan data acquisition, *m*/*z* 100–1000. Ion spectra were recorded from 5 to 40 eV with argon. Data processing was performed using LC/MS Solution 3.60.361 software (Shimadzu), while molecular formula prediction and elemental composition prediction were done using Formula Predictor and Accurate Mass Calculator software, respectively.

### 2.7. Quantitative Determination and PCA

The components of hesperidin and other four PMFs were detected at 283 nm and 330 nm in HPLC, respectively, which were calculated using the external standard method and expressed as mean ± SD. The contents of five flavonoids in all samples were first normalized by SPSS software (Version 16.0), and then principal component analysis was performed.

## 3. Results and Discussion

### 3.1. Antitussive and Expectorant Activities of MECRP

**Figure 1:**
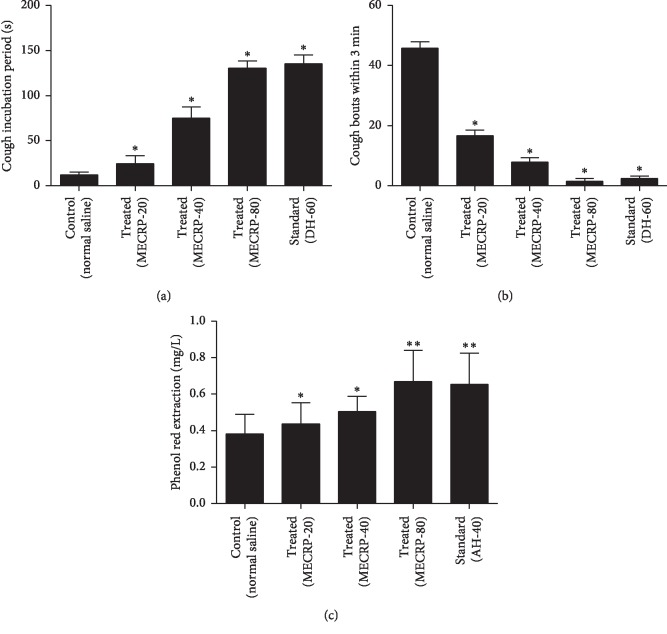
Effect of the MECRP on ammonia water-irritated cough and tracheal phenol red excretion in mice. (a) Incubation period of cough. (b) Bouts of cough. (c) Tracheal phenol red excretion. All values are expressed as mean ± SD (*n* = 10). ^*∗*^*P* < 0.05 and ^*∗∗*^*P* < 0.01 compared to the control group (one-way ANOVA followed by the LSD test).

The effects of the MECRP on ammonia water-irritated cough (Figures 1(a) and 1(b)) and tracheal phenol red excretion in mice are depicted in Figure 1(c). Statistical analysis showed significant differences between the control group and treated groups in the incubation period of cough (*P* < 0.05), bouts of cough within 3 minutes (*P* < 0.05), and tracheal phenol red excretion (*P* < 0.05). The test of ammonia water-irritated cough showed that the MECRP significantly prolonged the incubation period of cough and decreased the bouts of coughs in mice, possessing a good ability to relieve cough. In addition, the tracheal phenol red excretion test illustrated that the MECRP could effectively reduce sputum with the increase of the excretion of phenol red in the trachea. It has been well suggested that the MECRP exhibited excellent antitussive and expectorant activities in mice.

### 3.2. Similarity Analysis on “Activity Fingerprint”

**Figure 2:**
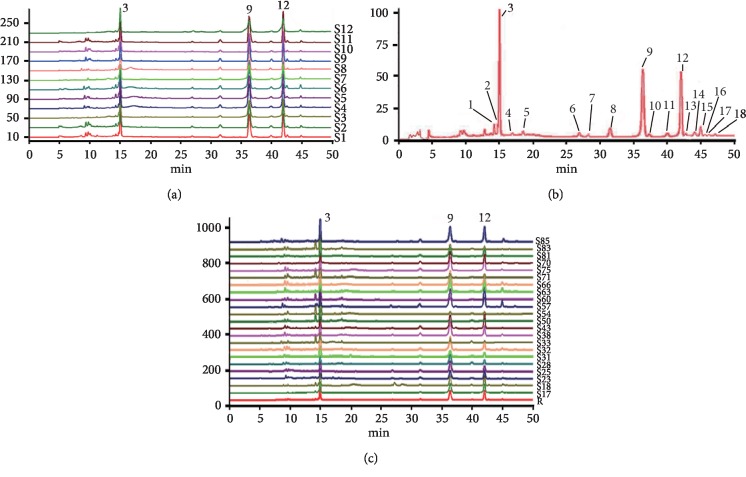
(a) GCP fingerprint of samples S1–S12. (b) GCP common pattern R. (c) Fingerprint of representative cultivars of CRP. Peaks 3, 9, and 12 are hesperidin, nobiletin, and tangeretin, respectively.

The GCP fingerprint (Figure 2(a)) was firstly established by twelve batches of traditional genuine herbs from Xinhui (S1–S12) due to their high quality. And a GCP common pattern (Figure 2(b)) was further generated through the characteristics of samples S1–S12. There were 18 common peaks determined in the GCP common pattern, of which peaks 3, 9, and 12 were identified as hesperidin, nobiletin, and tangeretin, respectively. According to statistical analysis, the common peak area of each sample (S1–S12) accounted for 97.276%–98.870% (>90%) of total peak areas. And all of the accuracy, repeatability, and stability results showed that the RSD of relative retention time and relative peak area of the 18 common peaks was below 3%, satisfying the demands of fingerprint.

The fingerprint of representative cultivars of CRP was finally established with the GCP common pattern (Figure 2(c)). In addition, the similarity evaluation results of 88 samples are shown in Table 1. The similarity values of samples S1–S12 compared to the GCP common pattern were all above 0.990, indicating that the *C*. *reticulata* “Chachi” samples from Xinhui were all of good quality uniformly. Moreover, the average similarity of the *C*. *reticulata* “Chachi” samples from other areas (S13–S22) was high (0.974), suggesting that there was little characteristic difference among *C*. *reticulata* “Chachi” samples. It was also seen that several cultivars of CRP showed high similarity to *C*. *reticulata* “Chachi,” such as *C*. *reticulata* “Shiyueju,” *C*. *reticulata* “Ponkan,” *C*. *reticulata* “Kinokuni,” *C*. *reticulata* “Yangshanju,” *C*. *reticulata* “Tribute,” *C*. *reticulata* “Bayueju,” *C*. *reticulata* “Suavissima,” *C*. *reticulata* “Wuyueju,” and *C*. *reticulata* “Nianju,” with the high average values of 0.962, 0.957, 0.953, 0.950, 0.949, 0.945, 0.942, 0.941, and 0.935, respectively. However, the cultivars of *C*. *reticulata* “Unshiu,” *C*. *reticulata* “Erythrosa,” *C*. *reticulata* “Yichangju,” *C*. *reticulata* “Qingjiangju,” *C*. *reticulata* “Linhaiju,” and *C*. *reticulata* “Xunwuju” showed little similarity to *C*. *reticulata* “Chachi,” with the low average similarity of 0.558, 0.539, 0.518, 0.504, 0.499, and 0.489, respectively.

### 3.3. Chemical Profiling on “Activity Fingerprint” by LC/MS-IT-TOF

**Figure 3:**
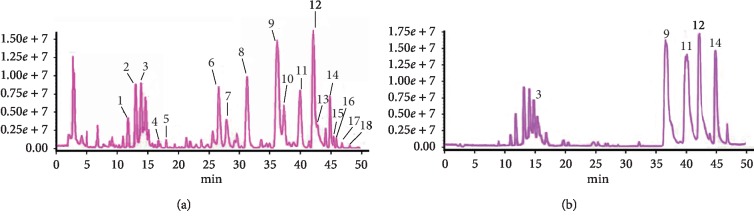
(a) Total ion chromatogram (TIC) of CRP in the positive mode. (b) TIC of mixed standard solution in the positive mode. Peaks 3, 9, 11, 12, and 14 are hesperidin, nobiletin, 3,5,6,7,8,3′,4′-heptamethoxyflavone, tangeretin, and 5-hydroxy-6,7,8,3′,4′-pentamethoxyflavone, respectively.

The technology of LC/MS-IT-TOF has been proved to be an effective method for identifying the unknown compounds in materials [21]. The total ion chromatogram (TIC) of CRP sample S1 (a) and mixed standard solution (b) in the positive mode is displayed in Figure 3. After chemical profiling, a total of 17 characteristic compounds, including 3 flavonoid glycosides and 14 PMFs, were identified according to the information of mass fragment ions and retention times, and the detailed information is illustrated in Table 2.

#### 3.3.1. Identification of Flavonoid Glycosides

Flavonoids are a kind of organic compounds with C6-C3-C6 as the parent nucleus. Among them, flavonoid glycosides are widely present in citrus, including flavone-O-glycosides and flavone-C-glycosides. The flavone-O-glycosides are generally characterized by attaching glycosidic substituents to the flavonoid skeleton via the C-7 hydroxyl group, while the flavone-C-glycosides directly linked glycosidic substituents to the C atoms on the flavone A ring [22].

Compound 2 displayed a quasimolecular ion at *m*/*z* 581, which formed a fragment ion at *m*/*z* 273 due to the loss of terminal Rha-Glc (308 Da). The MS spectrum also showed secondary fragment cracking by aglycon. For instance, the fragment at *m*/*z* 153 was produced by RDA cleavage, and the fragment at *m*/*z* 245 was formed corresponding to the flavonoid glycoside ion loss of CO (28 Da). Therefore, the compound was considered naringin according to the mass fragmentation information above [23].

Compound 3 was putatively identified as hesperidin because of the same mass spectrum and chromatographic characteristics with the reference standard. The high intensity ions at *m*/*z* 449 and 303 were observed on the removal of glucose (162 Da) and rhamnosylglucose (308 Da) from the precursor ion, respectively. An extra minor fragment at *m/z* 153 was yielded by the RDA cleavage of aglycone ion. Compound 4 showed the similar fragment information to compound 3 but was different in the relative abundances, which could be inferred as neohesperidin [24].

Compound 5 showed the [M + H]^+^ ion at *m*/*z* 595 in the first-order MS spectrum, suggesting the molecular formula of C_28_H_34_O_14_. A high-intensity ion at *m*/*z* 287 after losing neohesperidose (308 Da) was generated. This compound was tentatively inferred as poncirin after the comparison of its mass spectrum data [25].

#### 3.3.2. Identification of PMFs

PMFs are significant flavonoids particularly presented in citrus species, containing multiple methoxyl groups, low polarity, and planar structure [26]. With a basic unit of 222 Da, the molecular weights of PMFs vary depending on the amount of methoxyl and hydroxyl groups [27, 28].

Compound 9 was confirmed as nobiletin by comparison to the reference standard with the same fragmentation information. Nobiletin gave an [M + H]^+^ ion at *m*/*z* 403 and yielded fragment ions at *m*/*z* 388 ([M + H − CH_3_]^+^), 373 ([M + H − 2CH_3_]^+^), and 355 ([M + H − 2CH_3_ − H_2_O]^+^). Similarly, compound 11 was deduced as 3,5,6,7,8,3′,4′-heptamethoxyflavone by comparing to the reference standard with the [M + H]^+^ ion at *m*/*z* 433.

Compounds 6, 7, 8, 12, 14, and 15 were considered pentamethoxyflavones based on their molecular formula and fragment ions [29]. Comparing with a reference standard allowed us to identify compound 14 as 5-hydroxy-6,7,8,3,3′,4′-pentamethoxyflavone. It exhibited the parent ion [M + H]^+^ at *m*/*z* 389 and the product fragment ion at *m*/*z* 374 ([M + H − CH_3_]^+^), 359 ([M + H − 2CH_3_]^+^), and 341 ([M + H − 2CH_3_ − H_2_O]^+^). Compounds 6 and 8 were known as isosinensetin and sinensetin combining with the mass data of the previous research [30]. Compound 7 was presumed to be monohydroxy-pentamethoxyflavone based on the molecular formula of C_20_H_20_O_8_ and similar fragment of compound 14. Similarly, compound 15 was inferred to be dihydroxy-pentamethoxyflavone differed by the molecular weight of 16 of compound 7.

Compound 12 was identified as tangeretin by comparing the retention time and fragmentation information of the reference standard. Moreover, the fragmentation information of compound 17 was similar to that of compound 12 and differed by the molecular weight of 14. Combining with the fragment information previously reported [31], compound 17 was presumed to be 5-hydroxy-6,7,8,4′-tetramethoxyflavone.

### 3.4. Quality Evaluation by Quantitative Determination and PCA

Hesperidin and other four main PMFs selected from 17 characteristic compounds profiled above were determined, and the results are given in Table 1. The method validation showed a good linearity (*R*^2^ > 0.9990), and the RSDs of accuracy, repeatability, stability, and recovery were all below 3%, indicating that the assay method was feasible.

PCA is a well-known analysis method with the purpose of dimensionality reduction to transform multiple indicators into a few comprehensive indicators [32, 33]. Defining the contents of the five flavonoids as five characteristics, PCA was used to determine the correlation among samples from different cultivars in this research. The first two principal components were extracted by PCA (Table 3), accounting for 79.979% of the total variance (PC1 = 59.269% and PC2 = 20.710%). And the variable importance in projection (VIP) values of nobiletin, tangeretin, 3,5,6,7,8,3′,4′-heptamethoxyflavone, and 5-hydroxy-6,7,8,3′,4′-pentamethoxyflavone were 0.934, 0.965, −0.641, and 0.827, respectively, showing more contribution to PC1, while hesperidin contributed more to PC2 with the VIP value of 0.953. It was indicated that these five compounds were the characteristic chemicals distinguishing various cultivars of CRP; especially, the four PMFs contributed more to PC1, accounting for 59.269% of the total variance.

**Figure 4:**
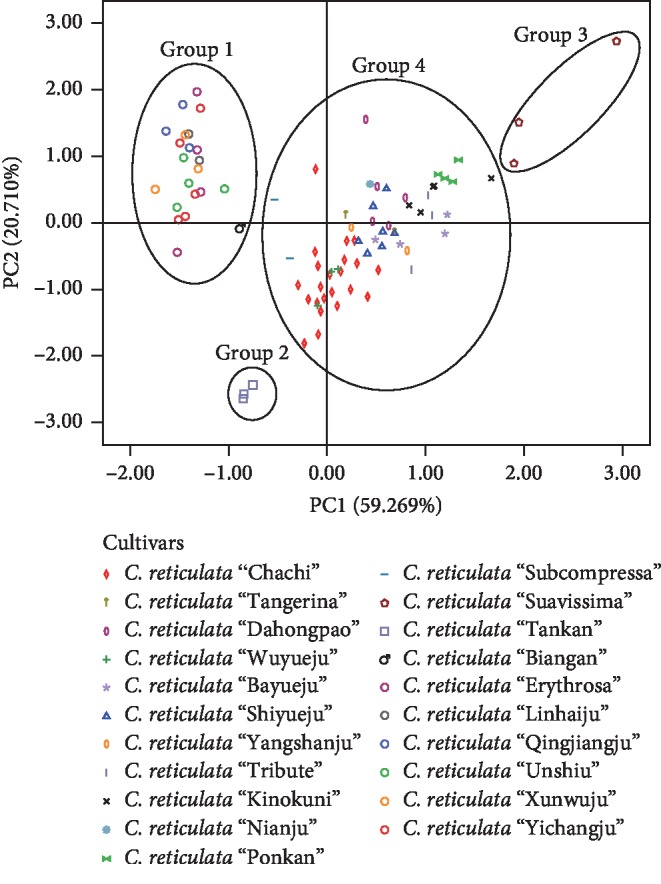
PCA results of CRP from different cultivars.

As the result of the PCA plot shows (Figure 4), 88 samples with 21cultivars were categorized into four groups. Group 1 contained *C. reticulata* “Unshiu,” *C. reticulata* “Erythrosa,” *C. reticulata* “Yichangju,” *C. reticulata* “Qingjiangju,” *C. reticulata* “Linhaiju,” *C. reticulata* “Xunwuju,” and *C*. *reticulata* “Biangan,” with the high content of hesperidin and low PMFs. *C. reticulata* “Tankan” was in group 2 due to its lower content of flavonoids, while *C. reticulata* “Suavissima” with a higher content of flavonoids was in group 3. Other eleven cultivars were clustered in group 4 with *C. reticulata* “Chachi,” showing parts of similarities and relationships with the traditional genuine herb. The result of PCA was approximately consistent with that of similarity analysis.

Since an LC/MS-IT-TOF was used, 17 characteristic compounds were finally profiled on the 18 common peaks at the “activity fingerprint,” and all of them were identified belonging to flavonoids, suggesting that there may be a potential relationship between the differences among varieties and flavonoid compositions. Then, hesperidin and four main PMFs were selected for quantitative determination and further analysis. And the PCA results confirmed that the differences among varieties were significantly correlated with flavonoids, not only including hesperidin, the single indicator for CRP quality control in Chinese Pharmacopoeia (version 2015) [1], but also including four main PMFs, such as nobiletin, tangeretin, 3,5,6,7,8,3′,4′-heptamethoxyflavone, and 5-hydroxy-6,7,8,3′,4′-pentamethoxyflavone. Therefore, we could not deny the importance of PMFs in CRP, and it is considerable to add PMFs as indicator components for CRP quality control and identification in Chinese Pharmacopoeia.

## 4. Conclusion

In the present study, an effective “activity fingerprint” on CRP from different cultivars involving antitussive and expectorant activity assays, similarity analysis, LC/MS-IT-TOF analysis, quantitative analysis, and PCA was perfectly established, which was proven to relate to pharmacological activity and conducted to find the characteristic chemicals distinguishing various cultivars of CRP for quality control. From the results above, the recorded cultivars of *C*. *reticulata* “Chachi,” *C*. *reticulata* “Tangerina,” and *C*. *reticulata* “Dahongpao” were quite similar to each other except for *C*. *reticulata* “Unshiu,” which indicated that the selection of the traditional medicinal herb of CRP was rational related to the high content of PMFs. In addition, there are some other cultivars significantly similar to *C*. *reticulata* “Chachi,” such as *C*. *reticulata* “Kinokuni,” *C*. *reticulata* “Yangshanju,” *C*. *reticulata* “Wuyueju,” and *C*. *reticulata* “Nianju,” and so on. Among them, it was worth noting that the cultivars of *C*. *reticulata* “Shiyueju,” *C*. *reticulata* “Ponkan,” *C*. *reticulata* “Tribute,” and *C*. *reticulata* “Bayueju” were rarely used for traditional medicine but exhibited a relatively high content of hesperidin and PMFs, even exceeding that of *C*. *reticulata* “Chachi.” Therefore, these four cultivars can be considered effective resources for the application of CRP, which provide a reference for citrus resource development.

## Figures and Tables

**Table 1:** Similarity evaluation and contents of five compounds of CRP collected from different regions in China.

No.	Cultivars	Specific origins	Harvest time	Storage (years)	Similarity	Average similarity	Contents of five flavonoids (mg/g, *n* = 3)^a,b^				
							1	2	3	4	5
S1	*C*. *reticulata* “Chachi” from the Xinhui region	Luokeng, Xinhui, Guangdong	2014.10.16	4	0.992	0.994	32.24 ± 0.01	3.52 ± 0.00	0.34 ± 0.00	2.43 ± 0.00	0.26 ± 0.00
S2		Yinhuwan, Xinhui, Guangdong	2014.10.14	4	0.999		29.62 ± 0.02	3.72 ± 0.00	0.35 ± 0.00	2.39 ± 0.00	0.28 ± 0.00
S3		Sanjiang, Xinhui, Guangdong	2014.11.18	4	0.998		27.59 ± 0.05	3.27 ± 0.01	0.27 ± 0.00	2.14 ± 0.00	0.22 ± 0.00
S4		Xiaogang, Xinhui, Guangdong	2014.10.24	4	0.994		28.67 ± 0.01	4.31 ± 0.00	0.38 ± 0.00	2.82 ± 0.00	0.26 ± 0.00
S5		Tianlu, Xinhui, Guangdong	2014.11.22	4	0.995		38.15 ± 0.02	3.82 ± 0.00	0.39 ± 0.00	2.41 ± 0.00	0.28 ± 0.00
S6		Yanan, Xinhui, Guangdong	2014.10.01	4	0.993		38.51 ± 0.03	5.03 ± 0.00	0.44 ± 0.00	3.14 ± 0.00	0.47 ± 0.00
S7		Siqian, Xinhui, Guangdong	2014.11.22	4	0.996		26.72 ± 0.03	2.63 ± 0.00	0.18 ± 0.00	1.73 ± 0.00	0.21 ± 0.00
S8		Shenlv, Xinhui, Guangdong	2015.10.11	3	0.991		18.14 ± 0.00	2.84 ± 0.00	0.28 ± 0.00	1.76 ± 0.00	0.16 ± 0.00
S9		Shuangshui, Xinhui, Guangdong	2014.11.02	4	0.992		21.38 ± 0.01	3.56 ± 0.00	0.37 ± 0.00	2.18 ± 0.00	0.24 ± 0.00
S10		Chakeng, Xinhui, Guangdong	2014.11.25	4	0.997		30.27 ± 0.02	2.27 ± 0.00	0.19 ± 0.00	1.67 ± 0.00	0.19 ± 0.00
S11		Nantan, Xinhui, Guangdong	2014.10.20	4	0.991		30.10 ± 0.01	3.92 ± 0.00	0.22 ± 0.00	3.18 ± 0.00	0.25 ± 0.00
S12		Qibao, Xinhui, Guangdong	2014.11.16	4	0.993		24.33 ± 0.01	2.90 ± 0.00	0.26 ± 0.00	2.16 ± 0.00	0.28 ± 0.00

S13	*C*. *reticulata* “Chachi” from other regions	Kaiping, Jiangmen, Guangdong	2014.09.26	4	0.974	0.974	43.09 ± 0.03	4.45 ± 0.00	0.34 ± 0.00	3.10 ± 0.00	0.36 ± 0.00
S14		Heshan, Jiangmen, Guangdong	2014.11.17	4	0.991		33.75 ± 0.03	3.30 ± 0.00	0.28 ± 0.00	2.70 ± 0.00	0.30 ± 0.00
S15		Gaoyao, Zhaoqing, Guangdong	2014.10.21	4	0.975		28.56 ± 0.01	2.93 ± 0.00	0.22 ± 0.00	2.75 ± 0.00	0.27 ± 0.00
S16		Gaoyao, Zhaoqing, Guangdong	2014.11.23	4	0.968		57.51 ± 0.02	2.67 ± 0.00	0.18 ± 0.00	2.66 ± 0.00	0.30 ± 0.00
S17		Wengyuanbazi, Shaoguan, Guangdong	2014.11.20	4	0.971		34.69 ± 0.01	3.86 ± 0.00	0.30 ± 0.00	2.78 ± 0.00	0.35 ± 0.00
S18		Longmen, Huizhou, Guangdong	2014.10.15	4	0.977		39.32 ± 0.01	2.91 ± 0.00	0.31 ± 0.00	2.40 ± 0.00	0.31 ± 0.00
S19		Longcheng, Huizhou, Guangdong	2014.10.18	4	0.979		36.47 ± 0.00	3.65 ± 0.00	0.23 ± 0.00	2.93 ± 0.00	0.34 ± 0.00
S20		Xinxing, Yunfu, Guangdong	2014.11.06	4	0.969		29.42 ± 0.04	4.57 ± 0.01	0.39 ± 0.00	3.58 ± 0.01	0.45 ± 0.00
S21		Cangwu, Wuzhou, Guangxi	2014.10.17	4	0.963		36.33 ± 0.10	5.25 ± 0.01	0.33 ± 0.00	3.98 ± 0.01	0.36 ± 0.00
S22		Lingshan, Qinzhou, Guangxi	2014.11.19	4	0.975		41.14 ± 0.00	4.07 ± 0.00	0.26 ± 0.00	3.16 ± 0.00	0.41 ± 0.00

S23	*C*. *reticulata* “Subcompressa”	Huangyan, Taizhou, Zhejiang	2014.12.10	4	0.924	0.885	40.63 ± 0.00	2.77 ± 0.00	0.48 ± 0.00	2.15 ± 0.00	0.27 ± 0.00
S24		Huangyan, Taizhou, Zhejiang	2014.11.20	4	0.846		53.07 ± 0.02	2.07 ± 0.00	0.34 ± 0.00	1.86 ± 0.00	0.19 ± 0.00
S25	*C*. *reticulata* “Wuyueju”	Sihui, Zhaoqing, Guangdong	2014.12.24	4	0.938	0.941	24.98 ± 0.02	3.54 ± 0.00	0.07 ± 0.00	1.38 ± 0.00	0.14 ± 0.00
S26		Deqing, Zhaoqing, Guangdong	2014.12.25	4	0.941		33.49 ± 0.01	4.44 ± 0.00	0.06 ± 0.00	1.73 ± 0.00	0.22 ± 0.00
S27		Sihui, Zhaoqing, Guangdong	2014.12.25	4	0.943		33.13 ± 0.00	4.22 ± 0.00	0.06 ± 0.00	1.68 ± 0.00	0.18 ± 0.00

S28	*C*. *reticulata* “Tankan”	Chenghai, Shantou, Guangdong	2015.02.18	3	0.873	0.871	17.81 ± 0.01	4.19 ± 0.00	1.07 ± 0.00	0.68 ± 0.00	0.27 ± 0.00
S29		Zhaoan, Zhangzhou, Fujian	2015.02.20	3	0.876		21.91 ± 0.00	5.55 ± 0.00	1.38 ± 0.00	1.03 ± 0.00	0.32 ± 0.00
S30		Puning, Jieyang, Guangdong	2015.08.15	3	0.866		21.48 ± 0.01	5.13 ± 0.00	1.15 ± 0.00	0.84 ± 0.00	0.31 ± 0.00

S31	*C*. *reticulata* “Biangan”	Guiping, Guigang, Guangxi	2014.12.23	4	0.765	0.765	47.57 ± 0.00	1.76 ± 0.00	0.38 ± 0.00	0.69 ± 0.00	0.08 ± 0.00

S32	*C*. *reticulata* “Nianju”	Longmen, Huizhou, Guangdong	2015.03.01	3	0.935	0.935	52.46 ± 0.01	5.54 ± 0.00	0.05 ± 0.00	2.21 ± 0.00	0.46 ± 0.00

S33	*C*. *reticulata* “Yichangju”	Zhijiang, Yichang, Hubei	2015.11.26	3	0.492	0.518	53.29 ± 0.01	0.18 ± 0.00	0.66 ± 0.00	0.10 ± 0.00	0.05 ± 0.00
S34		Honghu, Jingzhou, Hubei	2014.12.01	4	0.525		56.59 ± 0.08	0.24 ± 0.00	0.47 ± 0.00	0.18 ± 0.00	0.05 ± 0.00
S35		Yiling, Yichang, Hubei	2014.10.24	4	0.556		76.49 ± 0.06	0.62 ± 0.00	0.39 ± 0.00	0.41 ± 0.00	0.06 ± 0.00
S36		Yiling, Yichang, Hubei	2014.10.24	4	0.493		70.20 ± 0.03	0.23 ± 0.00	0.55 ± 0.00	0.13 ± 0.00	0.05 ± 0.00
S37		Dianjun, Yichang, Hubei	2014.11.27	4	0.525		53.08 ± 0.02	0.26 ± 0.00	0.59 ± 0.00	0.10 ± 0.00	0.04 ± 0.00

S38	*C*. *reticulata* “Dahongpao”	Chuanshan, Suining, Sichuan	2014.11.16	4	0.934	0.940	54.25 ± 0.02	6.64 ± 0.00	0.15 ± 0.00	2.36 ± 0.00	0.46 ± 0.00
S39		Shuangliu, Chengdu, Sichuan	2014.12.31	4	0.949		43.91 ± 0.09	5.32 ± 0.01	0.07 ± 0.00	2.48 ± 0.00	0.43 ± 0.00
S40		Fengjie, Chongqing, Sichuan	2014.12.17	4	0.929		70.68 ± 0.03	6.16 ± 0.00	0.18 ± 0.00	2.72 ± 0.00	0.45 ± 0.00
S41		Kai, Chongqing, Sichuan	2015.11.26	3	0.948		49.76 ± 0.02	6.79 ± 0.00	0.07 ± 0.00	3.00 ± 0.00	0.52 ± 0.00
S42		Anyue, Ziyang, Sichuan	2015.11.26	3	0.940		45.40 ± 0.03	6.90 ± 0.01	0.22 ± 0.00	2.62 ± 0.00	0.51 ± 0.00

S43	*C*. *reticulata* “Shiyueju”	Gaoyao, Zhaoqing, Guangdong	2014.12.25	4	0.958	0.962	54.18 ± 0.02	5.55 ± 0.00	0.09 ± 0.00	4.29 ± 0.00	0.22 ± 0.00
S44		Gaoyao, Zhaoqing, Guangdong	2014.12.24	4	0.962		42.86 ± 0.04	5.45 ± 0.01	0.05 ± 0.00	4.36 ± 0.00	0.20 ± 0.00
S45		Lipu, Guilin, Guangxi	2014.12.26	4	0.963		43.54 ± 0.00	5.23 ± 0.00	0.05 ± 0.00	4.05 ± 0.00	0.16 ± 0.00
S46		Guiping, Guigang, Guangxi	2014.12.24	4	0.957		40.48 ± 0.01	4.12 ± 0.00	0.03 ± 0.00	3.50 ± 0.00	0.11 ± 0.00
S47		Yingde, Qingyuan, Guangdong	2014.12.28	4	0.96		50.18 ± 0.01	5.39 ± 0.00	0.10 ± 0.00	3.69 ± 0.00	0.20 ± 0.00
S48		Yingde, Qingyuan, Guangdong	2014.12.25	4	0.965		38.74 ± 0.00	4.77 ± 0.00	0.10 ± 0.00	3.68 ± 0.00	0.14 ± 0.00
S49		Zengcheng, Guangzhou, Guangdong	2014.12.25	4	0.967		40.40 ± 0.01	5.54 ± 0.00	0.11 ± 0.00	3.73 ± 0.00	0.21 ± 0.00

S50	*C*. *reticulata* “Unshiu”	Daxiang, Shaoyang, Hunan	2014.10.28	4	0.473	0.558	65.73 ± 0.06	0.07 ± 0.00	0.49 ± 0.00	0.06 ± 0.00	0.05 ± 0.00
S51		Shigu, Hengyang, Hunan	2014.10.30	4	0.540		61.06 ± 0.05	0.42 ± 0.00	0.60 ± 0.00	0.30 ± 0.00	0.08 ± 0.00
S52		Zhangshu, Yichun, Jiangxi	2014.10.26	4	0.657		56.94 ± 0.05	0.94 ± 0.00	0.47 ± 0.00	0.69 ± 0.00	0.19 ± 0.00
S53		Lingui, Guilin, Guangxi	2014.11.28	4	0.563		57.07 ± 0.01	0.40 ± 0.00	0.69 ± 0.00	0.19 ± 0.00	0.02 ± 0.00
S54	*C*. *reticulata* “Xunwuju”	Sanbiao, Xunwu, Jiangxi	2014.11.01	4	0.485	0.489	71.18 ± 0.01	0.18 ± 0.00	0.51 ± 0.00	0.14 ± 0.00	0.09 ± 0.00
S55		Shuiyuan, Xunwu, Jiangx	2014.11.01	4	0.480		61.02 ± 0.02	0.09 ± 0.00	0.36 ± 0.00	0.09 ± 0.00	0.05 ± 0.00
S56		Luoshan, Xunwu, Jiangxi	2014.11.01	4	0.501		64.51 ± 0.09	0.32 ± 0.00	0.92 ± 0.00	0.19 ± 0.00	0.06 ± 0.00

S57	*C*. *reticulata* “Suavissima”	Pingyang, Wenzhou, Zhejiang	2014.11.01	4	0.945	0.942	75.54 ± 0.03	9.48 ± 0.02	0.11 ± 0.00	7.35 ± 0.02	1.97 ± 0.01
S58		Lucheng, Wenzhou, Zhejiang	2014.12.28	4	0.942		50.53 ± 0.03	7.30 ± 0.00	0.10 ± 0.00	5.22 ± 0.01	1.33 ± 0.00
S59		Ouhai, Wenzhou, Zhejiang	2014.12.01	4	0.939		60.55 ± 0.02	7.25 ± 0.00	0.09 ± 0.00	5.84 ± 0.00	1.32 ± 0.00

S60	*C*. *reticulata* “Qingjiangju”	Qingjiang, Zixing, Hunan	2014.11.30	4	0.485	0.504	77.43 ± 0.04	0.02 ± 0.00	0.41 ± 0.00	0.05 ± 0.00	0.05 ± 0.00
S61		Qingjiang, Zixing, Hunan	2014.10.28	4	0.506		67.26 ± 0.04	0.14 ± 0.00	0.43 ± 0.00	0.09 ± 0.00	0.05 ± 0.00
S62		Sishui, Zixing, Hunan	2014.10.28	4	0.521		75.31 ± 0.03	0.27 ± 0.00	0.69 ± 0.00	0.14 ± 0.00	0.05 ± 0.00

S63	*C*. *reticulata* “Tribute”	Deqing, Zhaoqing, Guangdong	2014.11.16	4	0.945	0.949	48.52 ± 0.01	5.59 ± 0.00	0.46 ± 0.00	3.95 ± 0.00	1.18 ± 0.00
S64		Deqing, Zhaoqing, Guangdong	2014.11.04	4	0.953		43.90 ± 0.01	5.65 ± 0.00	0.45 ± 0.00	4.10 ± 0.00	1.15 ± 0.00
S65		Deqing, Zhaoqing, Guangdong	2014.11.30	4	0.949		26.97 ± 0.00	4.46 ± 0.00	0.35 ± 0.00	2.89 ± 0.00	0.88 ± 0.00

S66	*C*. *reticulata* “Kinokuni”	Nanfeng, Fuzhou, Jiangxi	2014.10.24	4	0.951	0.953	46.90 ± 0.01	6.02 ± 0.00	0.22 ± 0.00	3.22 ± 0.00	0.78 ± 0.00
S67		Nanfeng, Fuzhou, Jiangxi	2014.11.20	4	0.953		54.85 ± 0.01	9.29 ± 0.00	0.30 ± 0.00	5.06 ± 0.00	1.01 ± 0.00
S68		Ningdu, Ganzhou, Jiangxi	2014.11.28	4	0.945		51.61 ± 0.00	6.40 ± 0.00	0.19 ± 0.00	4.42 ± 0.00	0.73 ± 0.00
S69		Nanfeng, Fuzhou, Jiangxi	2014.10.26	4	0.956		46.97 ± 0.01	7.05 ± 0.00	0.20 ± 0.00	3.58 ± 0.00	0.66 ± 0.00
S70		Ningdu, Ganzhou, Jiangxi	2014.12.03	4	0.961		51.73 ± 0.01	6.40 ± 0.00	0.19 ± 0.00	4.44 ± 0.00	0.74 ± 0.00

S71	*C*. *reticulata* “Erythrosa”	Mengquan, Shimen, Hunan	2014.10.29	4	0.571	0.539	80.52 ± 0.10	0.62 ± 0.00	0.40 ± 0.00	0.33 ± 0.00	0.08 ± 0.00
S72		Chujiang, Shimen, Hunan	2014.10.28	4	0.512		65.63 ± 0.05	0.16 ± 0.00	0.36 ± 0.00	0.11 ± 0.00	0.05 ± 0.00
S73		Zaoshi, Shimen, Hunan	2014.10.29	4	0.567		47.05 ± 0.0	0.36 ± 0.00	0.77 ± 0.00	0.19 ± 0.00	0.05 ± 0.00
S74		Xinguan, Shimen, Hunan	2015.10.21	3	0.507		55.50 ± 0.03	0.11 ± 0.00	0.37 ± 0.00	0.08 ± 0.00	0.05 ± 0.00

S75	*C*. *reticulata* “Bayueju”	Dongbei, Lianzhou, Guangdong	2014.11.29	4	0.941	0.945	41.15 ± 0.02	6.53 ± 0.01	0.04 ± 0.00	5.28 ± 0.01	0.42 ± 0.00
S76		Dongbei, Lianzhou, Guangdong	2014.11.23	4	0.943		38.62 ± 0.01	4.99 ± 0.00	0.04 ± 0.00	4.33 ± 0.00	0.30 ± 0.00
S77		Lipu, Guilin, Guangxi	2014.10.28	4	0.950		41.50 ± 0.03	5.34 ± 0.00	0.16 ± 0.00	3.24 ± 0.00	0.35 ± 0.00
S78		Sihui, Zhaoqing, Guangdong	2014.10.29	4	0.947		46.37 ± 0.01	7.13 ± 0.00	0.08 ± 0.00	5.13 ± 0.00	0.46 ± 0.00

S79	*C*. *reticulata* “Tangerina”	Minhou, Fuzhou, Fujian	2013.01.08	5	0.937	0.943	45.35 ± 0.02	4.29 ± 0.00	0.06 ± 0.00	2.06 ± 0.00	0.34 ± 0.00
S80		Fuqing, Fuzhou, Fujian	2015.12.30	3	0.949		43.92 ± 0.02	6.69 ± 0.00	0.17 ± 0.00	3.17 ± 0.00	0.42 ± 0.00

S81	*C*. *reticulata* “Yangshanju”	Chenxi, Wuzhou, Guangxi	2014.11.20	4	0.953	0.950	43.44 ± 0.02	4.38 ± 0.00	0.10 ± 0.00	2.62 ± 0.00	0.30 ± 0.00
S82		Yanan, Xinhui, Guangdong	2014.10.28	4	0.946		42.16 ± 0.02	7.65 ± 0.00	0.35 ± 0.00	3.74 ± 0.00	0.47 ± 0.00

S83	*C*. *reticulata* “Linhaiju”	Yongquan, Linhai, Zhejiang	2014.10.27	4	0.494	0.499	70.18 ± 0.06	0.10 ± 0.00	0.40 ± 0.00	0.06 ± 0.00	0.05 ± 0.00
S84		Yongquan, Linhai, Zhejiang	2014.10.29	4	0.503		62.52 ± 0.07	0.07 ± 0.00	0.33 ± 0.00	0.07 ± 0.00	0.05 ± 0.00
S85	*C*. *reticulata* “Ponkan”	Pinghe, Zhangzhou, Fujian	2015.02.23	3	0.946	0.957	49.34 ± 0.04	6.05 ± 0.00	0.10 ± 0.00	4.46 ± 0.01	0.92 ± 0.00
S86		Kecheng, Quzhou, Zhejiang	2015.02.17	3	0.967		54.22 ± 0.01	6.70 ± 0.01	0.05 ± 0.00	4.19 ± 0.00	0.94 ± 0.00
S87		Yongchun, Quanzhou, Fujian	2015.02.10	3	0.959		49.07 ± 0.02	5.46 ± 0.00	0.04 ± 0.00	4.27 ± 0.00	0.90 ± 0.00
S88		Dangyang, Yichang, Hubei	2015.02.15	3	0.954		49.23 ± 0.01	5.41 ± 0.00	0.02 ± 0.00	3.65 ± 0.00	0.96 ± 0.00

^a^1–5 denote the components of hesperidin, nobiletin, 3,5,6,7,8,3′,4′-heptamethoxyflavone, tangeretin, and 5-hydroxy-6,7,8, 3′,4′-pentamethoxyflavone, respectively. ^b^The content values of five flavonoids are shown as mean ± standard deviation (SD).

**Table 2:** Major chemical compounds of CRP identified by LC/MS-IT-TOF.

Peak no.	RT (min)	[M + H]^+^ (*m*/*z*)	Molecular formula	Fragment ions (m/z)	Identified compounds
2	14.746	581.1854	C_27_H_32_O_14_	563.1743, 545.1634, 401.1057, 339.0855, 315.0814, 273.0756, 263.0544, 245.0433, 231.0385, 219.0280, 195.0291, 179.0287, 153.0179, 135.0564	Naringin
3^a^	14.995	611.1956	C_28_H_34_O_15_	580.6021, 465.1423, 449.1420, 431.1322, 413.1220, 345.0960, 303.0855, 281.0644, 263.0533, 219.0288, 195.0278, 177.0539, 153.0179	Hesperidin
4	16.162	611.1957	C_28_H_34_O_15_	596.2038, 575.1750, 465.1420, 449.2690, 431.1318, 413.1227, 369.0966, 345.0954, 303.0864, 281.0647, 263.0539, 219.0288, 195.0280, 177.0540, 153.0179	Neohesperidin
5	18.506	595.2011	C_28_H_34_O_14_	449.1152, 433.1460, 397.1286, 379.1211, 365.0712, 287.0888, 256.0431, 224.1293, 195.0290, 183.0582, 165.0598	Poncirin
6	26.814	373.1273	C_20_H_20_O_7_	358.1033, 343.0800, 327.0501, 299.0504, 285.0613, 189.0301, 165.0171, 151.0752	Isosinensetin
7	28.119	389.1224	C_20_H_20_O_8_	374.0996, 360.0797, 359.0761, 341.0662, 331.0804, 328.0944, 313.0592, 211.0154, 193.0088	Monohydroxy-pentamethoxyflavone
8	31.411	373.1265	C_20_H_20_O_7_	357.0960, 343.0837, 339.0865, 329.1018, 327.0533, 313.0688, 312.1002, 297.0766, 269.0820, 181.0395	Sinensetin
9^a^	36.272	403.1386	C_21_H_22_O_8_	388.1121, 373.0921, 355.0809, 345.0944, 342.1099, 327.0657, 313.0708, 258.0526, 183.0296, 165.0519	Nobiletin
10	37.217	343.1140	C_19_H_18_O_6_	327.0402, 313.0709, 309.0764, 299.0914, 282.0887, 267.0650, 153.0186, 135.0642	Tetramethyl-O-scutellarin
11^a^	39.931	433.1510	C_22_H_24_O_9_	418.1239, 417.1167, 403.1003, 385.0903, 373.0529, 345.0600, 317.0655, 183.0288, 165.0188	3,5,6,7,8,3′,4′-Heptamethoxyflavone
12^a^	41.968	373.1281	C_20_H_20_O_7_	358.1050, 343.0812, 328.0579, 325.0725, 312.0993, 297.0761, 283.0589, 211.0256, 183.0292, 135.0442	Tangeretin
13	42.637	375.1458	C_19_H_18_O_8_	345.0606, 327.0507, 299.0558, 227.0550, 211.0613, 196.0361, 183.0287, 167.0343	Dihydroxy-tetramethoxyflavone
14^a^	44.908	389.1227	C_20_H_20_O_8_	374.0996, 359.0761, 356.0886,343.0440, 341.0662, 331.0804, 328.0944, 327.0862, 298.0473, 197.0092, 165.0758, 151.0520	5-Hydroxy-6,7,8,3′,4′-pentamethoxyflavone
15	45.577	405.1564	C_20_H_20_O_9_	375.1424, 345.1332, 329.1027, 241.0711, 226.0477, 211.0238, 165.0187	Dihydroxy-pentamethoxyflavone
16	46.073	419.1326	C_21_H_22_O_9_	404.1032, 389.0983, 371.0802, 357.0587, 163.0385	Monohydroxy-hexamethoxyflavone
17	47.053	359.1138	C_19_H_18_O_7_	344.0858, 329.0659, 326.1001, 311.0568, 298.0835, 210.1810	5-Hydroxy-6,7,8,4′-tetramethoxyflavone
18	48.439	375.1424	C_19_H_18_O_8_	345.0898, 241.0654, 226.0423, 211.0211, 183.0290	Dihydroxy-tetramethoxyflavone

^a^Confirmed in comparison with standard compounds.

**Table 3:** Component matrix of PCA.

Components	Variable importance in projection (VIP) values	
	PC1	PC2
Hesperidin	−0.255	0.953
Nobiletin	0.934	−0.121
3,5,6,7,8,3′,4′-Heptamethoxyflavone	−0.641	−0.247
Tangeretin	0.965	0.011
5-Hydroxy-6,7,8, 3′,4′-pentamethoxyflavone	0.827	0.227

2 components were extracted.

## Data Availability

The data used to support the findings of this study are included within the article.

## Supplementary Materials

Supplementary Materials Table of abbreviations and acronyms.
