# Supplementary material for: Construction and Chemical Profile on “Activity Fingerprint” of Citri Reticulatae Pericarpium from Different Cultivars Based on HPLC-UV, LC/MS-IT-TOF, and Principal Component Analysis
Source: Evid Based Complement Alternat Med. 2020 Mar 3;2020:4736152. doi: 10.1155/2020/4736152 (PMC7072102; doi:10.1155/2020/4736152)
Supplement: Supplementary Materials — Table of abbreviations and acronyms. [file 4736152.f1.doc]

**Supplementary material:**

Table of abbreviations and acronyms.

| **Full name** | **Abbreviation and acronym** |
| --- | --- |
| Citri Reticulatae Pericarpium | CRP |
| Chenpi | CP |
| Guang Chenpi | GCP |
| *Citrus reticulata* ‘Chachi’ | *C. reticulata* ‘Chachi’ |
| Methanol extraction of Citri Reticulatae Pericarpium | MECRP |
| Dextromethorphan hydrobromide | DH |
| Ambroxol hydrochloride | AH |
| High-performance liquid chromatography | HPLC |
| High-performance liquid chromatography combined with ion-trap and time-of-flight mass spectrometry | LC/MS-IT-TOF |
| Total ion chromatogram | TIC |
| Polymethoxyflavonoids | PMFs |
| Principal component analysis | PCA |
